# Dose–Response Effects of Oral Melatonin on Reproductive Parameters in Intact Male Pomeranian Dogs With Alopecia X: A Controlled Study

**DOI:** 10.1002/vms3.70410

**Published:** 2025-05-13

**Authors:** Arman Abdous, Mohammad Jokar, Mehran Farhoodi, Darioush Shirani, Fariborz Moayer

**Affiliations:** ^1^ Faculty of Veterinary Medicine, Ka.C. Islamic Azad University Karaj Iran; ^2^ Faculty of Veterinary Medicine University of Calgary, Calgary, AB T2N 1N4 Canada; ^3^ Department of Veterinary Clinical Sciences, Ka.C. Islamic Azad University Karaj Iran; ^4^ Department of Internal Medicine Faculty of Veterinary Medicine University of Tehran Tehran Iran; ^5^ Department of Veterinary Pathobiology, Ka.C. Islamic Azad University Karaj Iran

**Keywords:** Alopecia X, dogs, melatonin, semen quality

## Abstract

This study investigates the dose–response effects of oral melatonin on reproductive parameters in intact male Pomeranian dogs diagnosed with Alopecia X. Given melatonin's potential therapeutic effects on hair regrowth and reproductive health, this study aimed to evaluate its impact on semen quality, hormonal profiles and testicular haemodynamics. A total of 16 intact male Pomeranian dogs were randomly assigned to four groups: a control group receiving a placebo and three treatment groups receiving melatonin at low (0.1 mg/kg), medium (0.3 mg/kg) and high (0.5 mg/kg) doses, administered twice daily for 45 days. Reproductive parameters, including semen volume, sperm concentration, total sperm count, sperm motility and levels of testosterone and oestradiol 17‐ß, were assessed biweekly. Testicular haemodynamics were evaluated using pulsed Doppler ultrasonography. The results showed dose‐dependent improvements in semen quality and testicular blood flow, with the highest dose group demonstrating the most significant improvements. However, analysis of the testosterone‐to‐oestradiol ratio revealed a decrease in the treatment groups, highlighting a complex hormonal response. Although semen quality improved, the study's short duration may not have captured the full spermatogenesis cycle. These findings suggest that melatonin may enhance reproductive function in male dogs with Alopecia X, but further research is needed to clarify its long‐term endocrine effects and optimize treatment protocols.

## Graphical abstract

Oral melatonin administration at varying doses (0.1, 0.3 and 0.5 mg/kg) in male Pomeranian dogs with Alopecia X improves sperm quality, with dose‐dependent effects on semen concentration and motility. This suggests that melatonin may enhance reproductive health in dogs with Alopecia X.

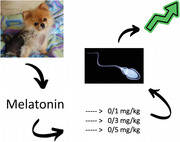

## 1 Introduction

Alopecia X is a dermatological condition primarily characterized by hair loss and arrest of the hair growth cycle, commonly affecting Pomeranian dogs and other Nordic breeds. Diagnosing Alopecia X is typically a process of exclusion, as no single diagnostic test definitively identifies the condition. Veterinarians often rely on ruling out other causes of hair loss, such as endocrine disorders, infections and nutritional deficiencies. Key diagnostic methods include clinical examination, hormonal assays to evaluate levels of thyroid hormones, ACTH (adrenocorticotropic hormone) and reproductive hormones (testosterone and oestradiol), as well as histopathological examination of skin biopsies. In some cases, an ultrasound scan may be performed to assess testicular size and function, providing insights into potential hormonal imbalances. The exact cause of Alopecia X remains unclear, though it is believed to be associated with hormonal imbalances, particularly involving the hypothalamic–pituitary–gonadal axis. This complex aetiology, combined with varying responses to treatments, makes Alopecia X a challenging condition for veterinarians. Among the therapeutic strategies explored, melatonin—a hormone primarily known for regulating circadian rhythms—has gained attention not only for its potential to treat alopecia but also for its broader effects on reproductive health (Welle 2023).

Melatonin is widely recognized for its biological functions, including antioxidant, anti‐inflammatory and endocrine‐regulating properties. It also plays a significant role in regulating vascular function, acting as a vasodilator by influencing the release of nitric oxide and modulating endothelial function. In veterinary medicine, melatonin is commonly prescribed to manage conditions such as anxiety, sleep disturbances and alopecia in canines. Although there is no universally agreed‐upon ‘standard dose’, clinical use of melatonin in dogs generally involves dosages ranging from 0.1 to 1.7 mg/kg, depending on the specific condition being treated. For instance, higher doses (1–1.7 mg/kg) are typically prescribed for conditions like seasonal alopecia or other hair disorders, whereas lower doses (0.1–0.3 mg/kg) are commonly used for promoting sleep or starting alopecia treatment. The dosage is often adjusted on the basis of the dog's clinical response, as individual variations can affect how the dog responds to melatonin treatment (Arendt and Aulinas 2022). Preliminary studies also suggest that melatonin may have beneficial effects on reproductive health in male dogs, potentially improving semen quality, enhancing testicular blood flow and modulating testosterone levels. However, further research is needed to establish the optimal dose for enhancing reproductive parameters (Sun et al. 2020). These findings are primarily based on standard dosages of melatonin, leaving a critical question unanswered: How do varying doses of melatonin impact reproductive outcomes in dogs?

The assessment of reproductive health in male dogs increasingly incorporates advanced imaging techniques such as Doppler ultrasonography, which provides valuable insights into testicular blood flow—a critical factor influencing spermatogenesis and overall semen quality (Velasco and Ruiz 2020). Previous research suggests that enhanced testicular blood flow is associated with improved sperm parameters, including concentration, motility and morphology. Given melatonin's known effects on vascular function, investigating its impact on testicular haemodynamics through Doppler ultrasonography offers a novel approach to understanding the underlying mechanisms by which melatonin may influence reproductive health.

The aim of this study is to evaluate the dose–response relationship of oral melatonin on reproductive parameters in intact male Pomeranian dogs, with a particular focus on semen quality, hormonal profiles and testicular haemodynamics. The rationale for this investigation is grounded in the understanding that **Alopecia X** is associated with hormonal imbalances, particularly within the hypothalamic–pituitary–gonadal axis, which may affect not only hair growth but also reproductive health. Previous research has shown that melatonin can positively influence testicular blood flow and spermatogenesis, improving semen quality and modulating hormonal levels, which makes it a promising candidate for enhancing reproductive health in affected dogs (Salama et al. 2022). By administering low, medium and high doses of melatonin, this research seeks to identify the optimal dosage for improving both reproductive parameters and hair regrowth. The findings will contribute to a deeper understanding of melatonin's role in canine reproduction and provide evidence‐based guidance for veterinarians treating dogs with reproductive and endocrine‐related disorders such as Alopecia X.

## 2 Methods

### 2.1 Study Design and Animals

This study was designed as a randomized, controlled trial to evaluate the dose–response effects of oral melatonin on reproductive parameters in intact male Pomeranian dogs diagnosed with Alopecia X. A total of 16 clinically healthy, intact male Pomeranian dogs (BW: 3.5 ± 0.5 kg, age: 4 ± 0.5 years) were selected for the study.

The diagnosis of Alopecia X was established through a combination of clinical examination and exclusion of other dermatological and endocrine conditions. The selected dogs exhibited symmetrical non‐inflammatory alopecia primarily affecting the trunk, with hyperpigmentation of the skin and coat cycle arrest. To confirm Alopecia X and rule out other potential causes of hair loss, all dogs underwent hormonal testing, including thyroid function assessment (total thyroxine, free thyroxine and thyroid‐stimulating hormone levels) to exclude hypothyroidism and ACTH stimulation testing to rule out hyperadrenocorticism. Reproductive hormone analysis was performed to measure baseline testosterone and oestradiol concentrations. Additionally, histopathological evaluation of skin biopsies was conducted, revealing characteristic findings such as follicular atrophy, epidermal hyperpigmentation and the absence of inflammatory infiltrates, consistent with Alopecia X.

Before melatonin treatment, all males underwent monthly semen collection to establish baseline reproductive parameters. The dogs were housed indoors, provided with daily exercise, a commercial diet and unrestricted access to water. Each dog was clinically examined, and testicular ultrasonography was performed to evaluate reproductive organ morphology and function.

**FIGURE 1:**
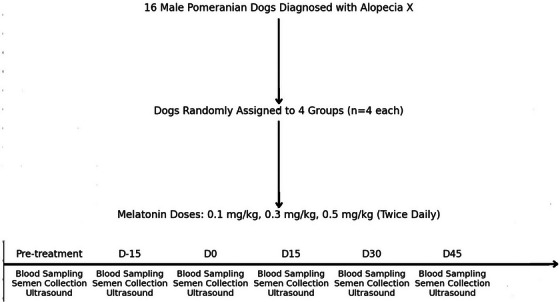
Experimental design flowchart showing the schedule of periodic assessments, including melatonin administration (D0) and evaluations at D‐15, D0, D15, D30 and D45 in 16 male Pomeranian dogs diagnosed with Alopecia X.

The dogs were randomly assigned to one of four experimental groups, with each group consisting of four dogs (*n* = 4 per group). The groups included a control group (placebo) and three treatment groups receiving melatonin at doses of 0.1, 0.3 and 0.5 mg/kg, respectively. The study was conducted over a 45‐day period, with reproductive assessments performed biweekly (Figure 1).

#### 2.1.1 Melatonin Administration

Melatonin tablets were administered orally in the form of an active pharmaceutical ingredient (API) formulation (Darou Pakhsh Pharmaceutical Manufacturing Co.) at doses of 0.1 mg/kg (low dose), 0.3 mg/kg (medium dose) or 0.5 mg/kg (high dose), twice daily (every 12 h). The treatment groups received melatonin in the morning (9:00 AM) and evening (9:00 PM), whereas the control group received a placebo (150 mg of lactose powder) at the same frequency. The dosage selection was based on a review of existing literature and was further refined following a preliminary pilot study to ensure safety and tolerability. To maintain consistency in administration and minimize variability in absorption, both melatonin and placebo were incorporated into the dogs’ food. The treatment was continued for a total duration of 6 weeks.

#### 2.1.2 Semen Collection and Analysis

In the presence of an oestrous bitch, the procedure involved rubbing the male dog's penis until a partial erection, followed by inducing a complete erection. The first two semen fractions were collected for volume determination (in mL) and sent to the lab. Samples, collected every 15 days, were stored in falcon tubes. Canine semen volume was measured using a calibrated tube. Sperm cell concentration and total count were determined with a light microscope and a haemocytometer. Sperm motility, estimated immediately after collection, was rounded to the nearest 5% using a phase‐contrast microscope (200× magnification) with a heated stage (Christensen and Meyers 2023).

#### 2.1.3 Hormonal Analysis

Blood samples were drawn from the jugular vein, before the Doppler ultrasonography examination, and centrifuged at 3000 × *g* for 15 min. Plasma samples were kept at −20°C before hormonal analysis. The samples were used for the assessment of testosterone (T) and oestradiol 17‐ß (E2). The quantitative analyses of testosterone and 7β‐oestradiol concentrations were performed in a dedicated RIA laboratory. For testosterone, a commercial testosterone kit from Immunotech, Beckman Coulter Ltd., Prague, Czech Republic, was employed, with the manufacturer indicating an assay sensitivity of 0.02 ng/mL (analytical sensitivity) and 0.08 ng/mL (functional sensitivity). The intra‐ and inter‐assay coefficients of variation were maintained at levels less than or equal to 5.6% and 15%, respectively. In the case of 7β‐oestradiol, the analysis took place in the same specialized RIA laboratory using a chemiluminescent microparticle immunoassay (ARCHITECT Estradiol, Abbott Laboratories, Abbott Park, Illinois, USA). The manufacturer specified an assay sensitivity of ≤10 pg/mL (analytical sensitivity) and ≤25 pg/mL (functional sensitivity), with coefficients of variation adhering to the criterion of less than or equal to 20%.

#### 2.1.4 Testicular Haemodynamics Assessment

**FIGURE 2:**
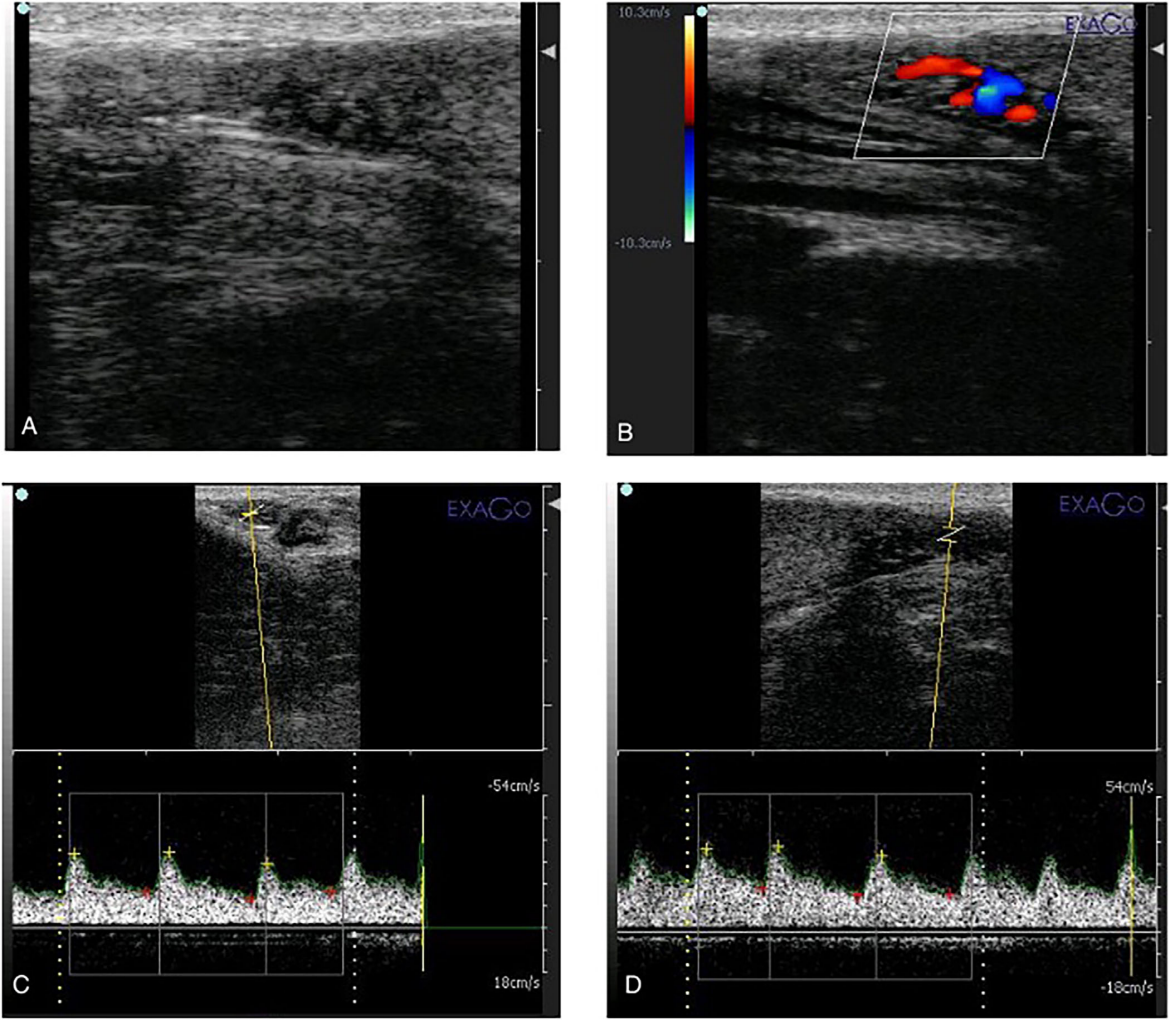
B‐mode (A) and colour Doppler (B) and power Doppler (C and D) ultrasonograms showing the pampiniform plexus area in canine testicles.

A bored certified radiologist performed all ultrasound examinations with a linear array probe. The settings of the Doppler device were developed as follows: The frequency range was 5.7–10 MHz, the pulse repetition frequency was 4000 kHz, and the angle of insonation was 45° ± 5°. The wall filter was 150, and the window size was 1 (Gloria et al. 2020). The Doppler assessment measured parameters such as peak systolic velocity (PSV), end‐diastolic velocity (EDV), Pulsatility Index (PI) and Resistive Index (RI) of the testicular arteries. These parameters were used to evaluate blood flow to the testes, which is known to correlate with spermatogenic activity and semen quality (Luño et al. 2020). The identification of the distal branch of the testicular artery was carried out through the analysis of image‐specific waves (Figure 2).

#### 2.1.5 Statistical Analysis

Data were analysed using SPSS software (version 25.0). The normality of data distribution was assessed using the Shapiro–Wilk test. A repeated measures ANOVA was performed to evaluate the effects of different melatonin doses on semen parameters, hormonal levels and testicular haemodynamics over time. When significant differences were detected, post hoc comparisons were conducted using the Tukey HSD test. A *p* value less than 0.05 was considered statistically significant, and a *p* value less than 0.01 was considered highly significant.

## 3 Results

The administration of melatonin at varying doses had a dose‐dependent effect on semen parameters in intact male Pomeranian dogs (Table 1). Semen volume increased slightly across all treatment groups compared to the control, with the most significant increase observed in the high‐dose group (0.5 mg/kg). The mean semen volume for the high‐dose group was 6.05 ± 0.02 mL, compared to the control group (4.35 ± 0.01 mL, *p* = 0.002), whereas the differences between the low‐dose (0.1 mg/kg; 4.20 ± 0.02 mL, *p* = 0.72) and medium‐dose (0.3 mg/kg; 4.25 ± 0.02 mL, *p* = 0.65) groups showed no statistically significant differences.

**TABLE 1:** Effects of melatonin doses on reproductive parameters and hormone levels over 45 days.

	Day 0				Day 15				Day 30				Day 45			
		Melatonin				Melatonin				Melatonin				Melatonin		
	Control	0.1 mg/kg	0.3 mg/kg	0.5 mg/kg	Control	0.1 mg/kg	0.3 mg/kg	0.5 mg/kg	Control	0.1 mg/kg	0.3 mg/kg	0.5 mg/kg	Control	0.1 mg/kg	0.3 mg/kg	0.5 mg/kg
Semen volume (mL)	4.35 ± 0.01	4.20 ± 0.02	4.25 ± 0.02	4.30 ± 0.02	4.95 ± 0.01	4.25 ± 0.01	4.31 ± 0.01	4.38 ± 0.02	4.88 ± 0.02	5.25 ± 0.02	5.92 ± 0.01	6.05 ± 0.02	4.96 ± 0.03	5.50 ± 0.02	6.02 ± 0.01	6.20 ± 0.02^**^
Concentration (*106/mL)	172 ± 26.3	179 ± 28.5	181 ± 31.2	184 ± 30.1	191 ± 15.5	220 ± 20.4	255 ± 30.2	260 ± 35.5	201 ± 25.3	240 ± 28.5	295 ± 29.8^*^	310 ± 31.0^*^	211 ± 40.9	240 ± 45.0	281 ± 65	300 ± 67
Total sperm (*106/ejaculation)	750 ± 22.2	760 ± 24.1	769 ± 25.1	780 ± 26.3	945 ± 45.6	1005 ± 50.3	1099 ± 5.5	1150 ± 60.7	980 ± 88	1450 ± 50	1746 ± 42	1850 ± 55	1046 ± 56	1420 ± 58	1691 ± 62^*^	1800 ± 64^**^
Motility%	67.32 ± 2.21	66.78 ± 2.35	67.12 ± 4.89	67.45 ± 3.10	72.35 ± 4.22	70.5 ± 3.5	68.71 ± 2.1	70.0 ± 2.8	72.58 ± 3.28	78 ± 3.0	82 ± 2.21	84 ± 2.5	77 ± 3.21	79 ± 3.0	82 ± 3.21*	84 ± 3.3^**^
Testosterone (ng/mL)	6.85 ± 1.08	6.95 ± 1.12	7.02 ± 2.02	7.10 ± 1.89	6.8 ± 4.07	7.0 ± 3.5	7.4 ± 1.08	7.6 ± 1.2	7.1 ± 1.02	7.8 ± 1.0	8.15 ± 0.05	8.3 ± 0.1	6.98 ± 1.05	7.5 ± 1.1	8.57 ± 0.08	8.9 ± 0.09
Oestradiol 17‐ß (pg/mL)	29.78 ± 5.66	29.98 ± 6.02	30.12 ± 7.08	30.25 ± 7.15	28.89 ± 8.12	35.15 ± 7.5	41.25 ± 9.02	44.10 ± 9.5	29.02 ± 9.11	40.0 ± 8.0	49.88 ± 8.32	52.5 ± 8.5	28.98 ± 11.21	40.0 ± 8.0	55.52 ± 7.08	58.0 ± 7.2
Supra‐testicular A PI	1.18 ± 0.02	1.20 ± 0.01	1.22 ± 0.01	1.23 ± 0.01	1.15 ± 0.01	1.16 ± 0.02	1.15 ± 0.02	1.13 ± 0.03	1.1 ± 0.03	1.05 ± 0.02	1.03 ± 0.02	1.02 ± 0.03	1.14 ± 0.01	1.10 ± 0.01	1 ± 0.01^*^	0.95 ± 0.02^**^
Supra‐testicular A RI	0.87 ± 0.01	0.90 ± 0.02	0.92 ± 0.02	0.93 ± 0.02	0.87 ± 0.02	0.86 ± 0.01	0.84 ± 0.01	0.83 ± 0.02	0.88 ± 0.01	0.70 ± 0.02	0.58 ± 0.01	0.55 ± 0.01	0.87 ± 0.01	0.70 ± 0.02	0.55 ± 0.01^*^	0.50 ± 0.02^**^

*Note*: Data are presented as mean ± standard deviation. **Asterisks** (*) indicate statistically significant differences at *p* < 0.05, and double asterisks (**) indicate highly significant differences at *p* < 0.01, compared to the control group.

Abbreviations: PI, Pulsatility Index; RI, Resistive Index.

Sperm concentration improved significantly in the medium (0.3 mg/kg; 295 ± 29.8 × 10^6/mL, *p* = 0.039) and high‐dose (0.5 mg/kg; 310 ± 31.0 × 10^6/mL, *p* = 0.035) groups compared to the control group (172 ± 26.3 × 10^6/mL). The low‐dose group (0.1 mg/kg; 179 ± 28.5 × 10^6/mL, *p* = 0.88) showed a moderate but non‐significant increase.

Total sperm count was significantly higher in the high‐dose group (1850 ± 55 × 10^6/ejaculation, *p* = 0.003) compared to the control group (750 ± 22.2 × 10^6/ejaculation). The medium‐dose group also showed a significant increase (1746 ± 42 × 10^6/ejaculation, *p* = 0.045), whereas the low‐dose group exhibited a non‐significant improvement (760 ± 24.1 × 10^6/ejaculation, *p* = 0.83).

**FIGURE 3:**
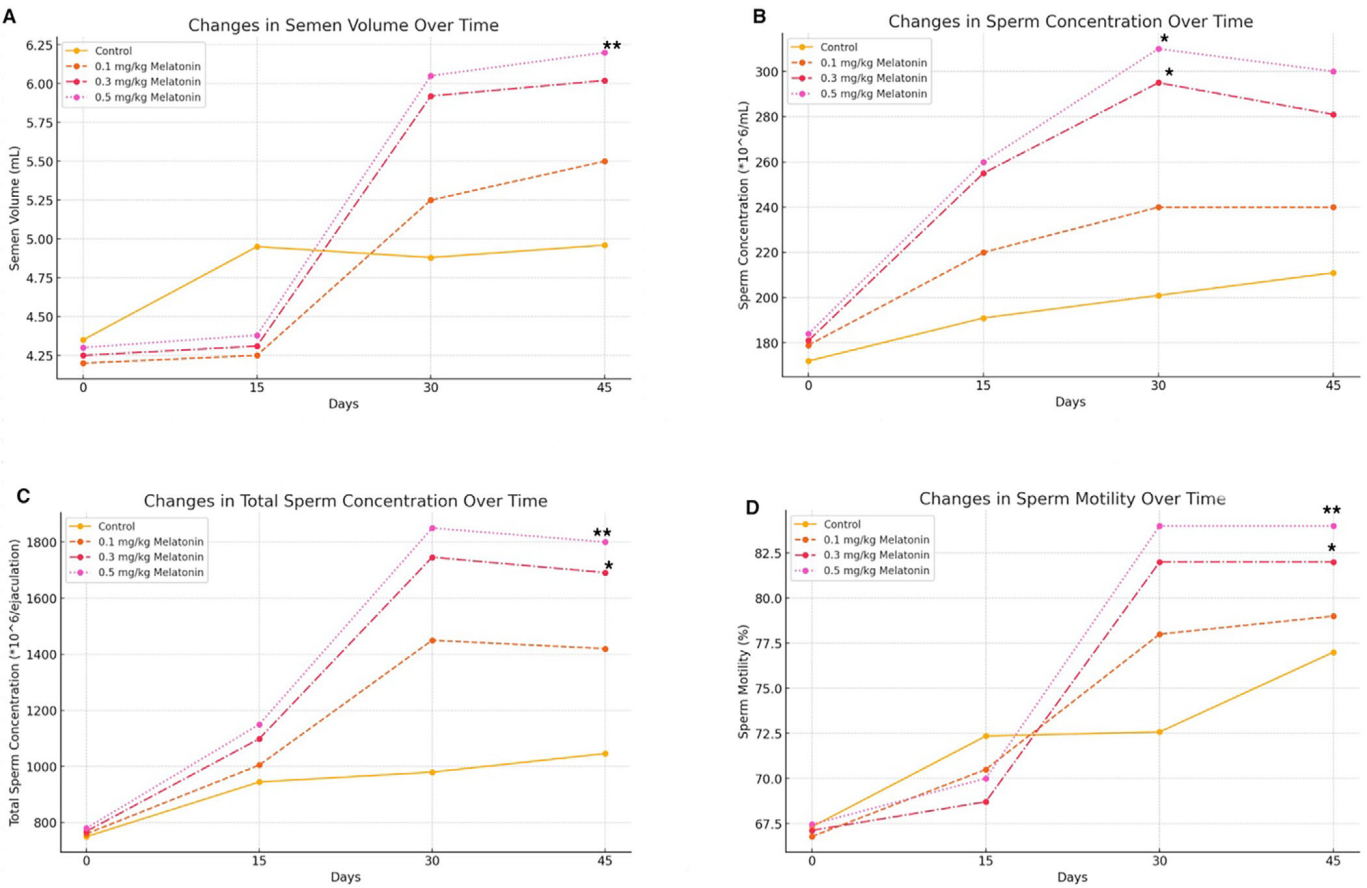
Effects of different doses of melatonin on reproductive parameters over time. (A) Semen volume (mL) at different time points. (B) Sperm concentration (*10^6/mL) over time. (C) Total sperm concentration (*10^6/ejaculation) at different time points. (D) Sperm motility (%) over time. Asterisks (*) indicate statistically significant differences at *p* < 0.05, and double asterisks (**) indicate highly significant differences at *p* < 0.01, compared to the control group.

Sperm motility improved in all treatment groups, with the high‐dose group demonstrating the most pronounced effect, resulting in a significant increase in motility (84% ± 2.5%, *p* = 0.004) compared to the control (67.32% ± 2.21%). The medium‐dose group also showed significant improvements in motility (82% ± 3.21%, *p* = 0.021), whereas the low‐dose group had a non‐significant increase (72.58% ± 3.28%, *p* = 0.12) (Figure 3).

Hormonal responses revealed dose‐dependent but non‐significant variations. Testosterone levels slightly increased in the high‐dose group (8.9 ± 0.09 ng/mL, *p* = 0.10) compared to the control (6.85 ± 1.08 ng/mL). The low‐dose (6.95 ± 1.12 ng/mL, *p* = 0.91) and medium‐dose (7.0 ± 3.5 ng/mL, *p* = 0.87) groups showed minimal, non‐significant variations compared to the control.

**FIGURE 4:**
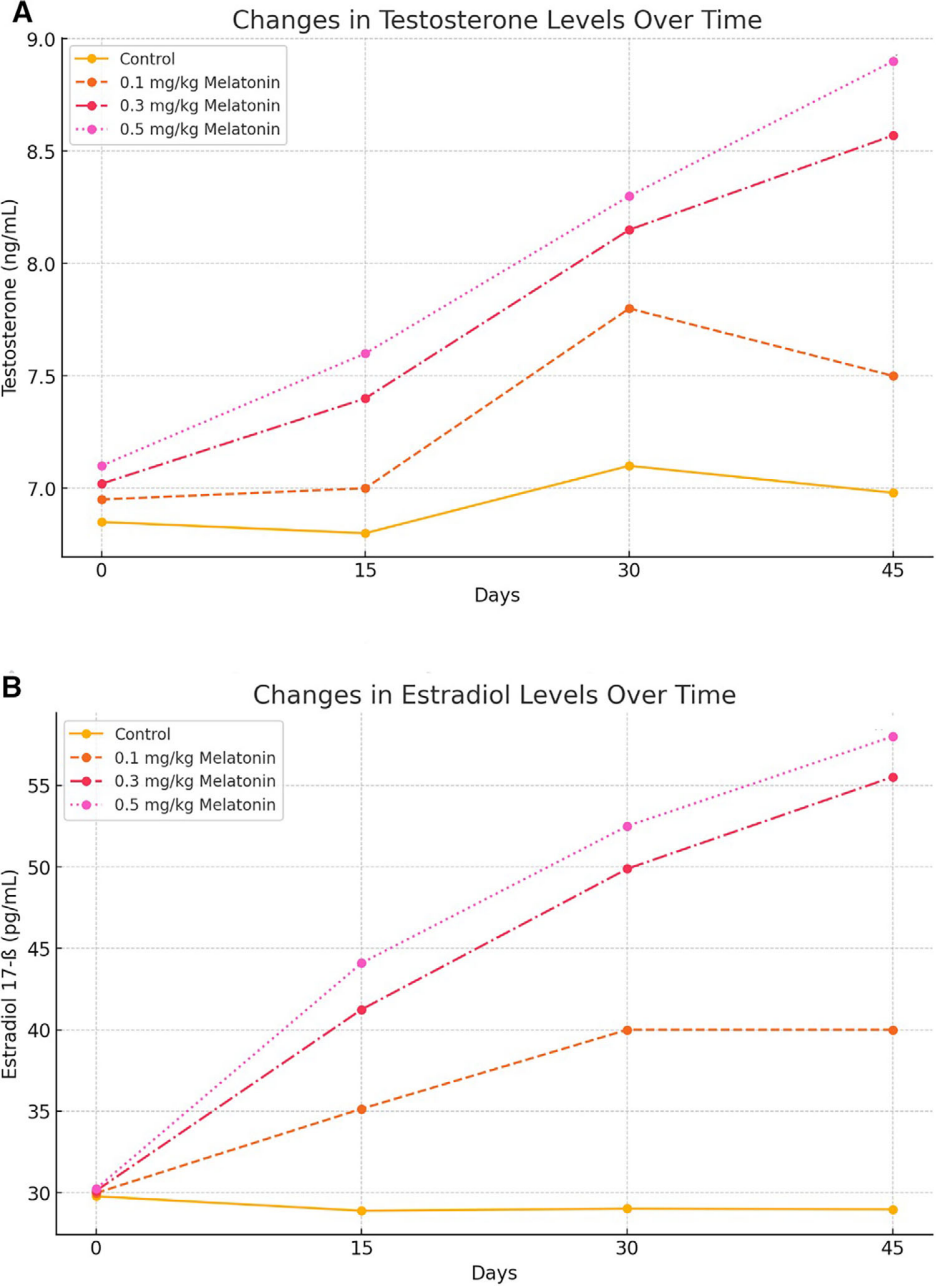
Hormonal changes in response to melatonin treatment over time. (A) Testosterone levels (ng/mL) over the study period. (B) Oestradiol 17‐ß levels (pg/mL) over time.

Oestradiol 17‐ß levels showed a modest but non‐significant increase in the high‐dose group (58.0 ± 7.2 pg/mL, *p* = 0.06) compared to the control (29.78 ± 5.66 pg/mL). The low‐dose (29.98 ± 6.02 pg/mL, *p* = 0.95) and medium‐dose groups (35.15 ± 7.5 pg/mL, *p* = 0.54) also did not differ significantly from the control (Figure 4).

**FIGURE 5:**
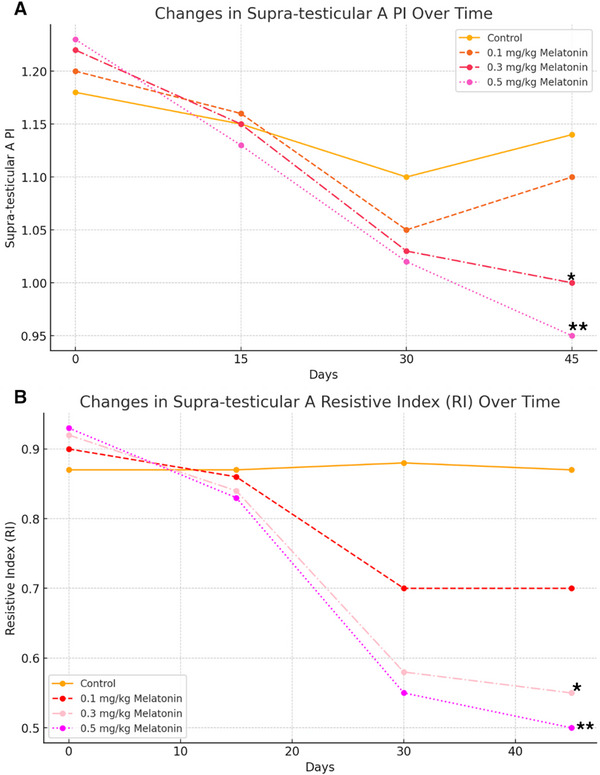
Effects of melatonin on testicular blood flow indices over time. (A) Pulsatility Index (PI) over the course of the study. (B) Resistive Index (RI) over the study period. Asterisks (*) indicate statistically significant differences at *p* < 0.05, and double asterisks (**) indicate highly significant differences at *p* < 0.01, compared to the control group.

Doppler ultrasonography showed dose‐dependent improvements in testicular blood flow as indicated by changes in the PI and RI. On Day 45, the PI significantly decreased in the high‐dose group (0.5 mg/kg; 0.95 ± 0.02, *p* = 0.004) and medium‐dose group (0.3 mg/kg; 1.00 ± 0.01, *p* = 0.035) compared to the control group (1.14 ± 0.01). However, the PI reduction in the low‐dose group (0.1 mg/kg; 1.10 ± 0.01, *p* = 0.12) was not statistically significant (Figure 5).

Similarly, RI values significantly decreased in both the high‐dose group (0.5 mg/kg; 0.50 ± 0.02, *p* = 0.002) and medium‐dose group (0.3 mg/kg; 0.55 ± 0.01, *p* = 0.042) compared to the control group (0.87 ± 0.01) on Day 45. The low‐dose group showed a non‐significant reduction (0.70 ± 0.02, *p* = 0.88) (Figure 5).

### 3.1 Testosterone to Oestradiol Ratio

The testosterone to oestradiol ratios were calculated for all groups at each time point (Days 0, 15, 30 and 45). In the control group, the ratio remained relatively stable throughout the study period, with a consistent value of approximately 0.23. In the melatonin‐treated groups, the ratios showed minor variations. Specifically, the 0.1 and 0.3 mg/kg groups maintained ratios ranging from 0.23 to 0.18, whereas the 0.5 mg/kg group exhibited a slight decrease in the ratio from 0.24 to 0.17 over time. Despite these changes, no statistically significant differences were observed between the groups (Table 2).

**TABLE 2:** Displays the testosterone to oestradiol ratios for the control and melatonin‐treated groups at each time point (Days 0, 15, 30 and 45).

Day	Control	0.1 mg/kg	0.3 mg/kg	0.5 mg/kg
0	0.230020148	0.231821214	0.233067729	0.235375562
15	0.235375562	0.199146515	0.199146515	0.199146515
30	0.234710744	0.179393939	0.179393939	0.179393939
45	0.244658856	0.172335601	0.172335601	0.172335601

## 4 Discussion

The results of this study underscore the significant dose‐dependent effects of oral melatonin on reproductive parameters in intact male Pomeranian dogs diagnosed with Alopecia X. Both studies consistently demonstrate that higher doses of melatonin (0.5 mg/kg) result in the most substantial improvements in semen quality, particularly in sperm concentration, total sperm count and motility. These findings align with previous research, such as that by Sun et al. (2020), which highlights melatonin's antioxidant properties and its role in enhancing male reproductive function by modulating the hypothalamic–pituitary–gonadal axis and protecting against oxidative stress‐induced DNA damage (Sun et al. 2020; Zdunczyk and Domoslawska 2022).

Interestingly, the effects of melatonin on serum testosterone and oestradiol 17‐ß levels were modest, with slight increases observed in the high‐dose groups, although the changes were not statistically significant. This suggests that melatonin's primary mode of action may be localized within the testes, particularly in its interaction with receptors on spermatogonial cells. Deng et al. (2018) suggest that melatonin may act directly at the testicular level, potentially affecting spermatogenesis through its antioxidative action and influence on the local hormonal environment. This contrasts with previous studies, such as those by Alvarez‐García et al. (2013) and Rosa et al. (2000), which reported more significant hormonal alterations (Alvarez‐García et al. 2013; Rosa et al. 2000). The differences in our findings may be attributed to variations in study design, species, or the duration of treatment. Furthermore, the minimal hormonal variation observed in the low‐ and medium‐dose groups further supports the hypothesis that melatonin's beneficial effects on reproductive health are more likely mediated at the testicular level rather than through systemic changes in serum hormone levels.

A significant aspect of our study was the use of Doppler ultrasonography to assess testicular blood flow, which provided valuable insights into melatonin's vascular effects (Zelli et al. 2013). Notably, significant improvements in testicular haemodynamics, particularly in PI and RI, were observed in the high‐dose groups. These findings are consistent with previous research by El‐Shalofy et al. (2022) and El‐Sherbiny et al. (2022), who suggested that melatonin may enhance testicular blood flow by reducing vascular resistance. This improvement in vascular parameters is likely crucial for optimizing spermatogenesis, as adequate blood flow supports the delivery of oxygen and nutrients to testicular tissues, enhancing sperm production and overall semen quality (El‐Sherbiny et al. 2022; El‐Shalofy et al. 2022; Samir et al. 2020).

In addition to these findings, the calculation of the testosterone to oestradiol ratio provided further insights into the potential impact of melatonin on reproductive health (Song et al. 2021). Throughout the study, the testosterone to oestradiol ratio remained relatively stable across both the control and melatonin‐treated groups. The slight decrease observed in the 0.5 mg/kg group, from 0.24 to 0.17, was not associated with any significant hormonal imbalances that could disrupt spermatogenesis. The ratios in the melatonin‐treated groups were consistent with those in the control group, suggesting that the observed improvements in semen quality were not a result of abnormal oestrogen increases that could negatively affect sperm production. The correlation between the testosterone to oestradiol ratio and semen quality parameters was not strong, indicating that melatonin's effects on semen quality may not be solely driven by changes in the testosterone to oestradiol balance.

When comparing the different doses of melatonin, the dose‐dependent improvements in semen parameters suggest that higher doses, such as 0.5 mg/kg, are more effective in optimizing semen quality. However, despite these improvements, the lack of significant changes in the testosterone‐to‐oestradiol ratio, even at higher doses, raises important questions regarding the long‐term safety and efficacy of high‐dose melatonin. Prolonged high‐dose administration may affect hormonal feedback mechanisms and other endocrine functions. Furthermore, our study provides a deeper understanding of melatonin's mechanisms, suggesting that melatonin may exert its effects through antioxidant properties and by influencing the hypothalamic–pituitary–gonadal axis, as well as potentially acting locally within the testes (Mogheiseh et al. 2022). The enhancement of testicular blood flow could also play a crucial role in supporting healthy spermatogenesis. However, further research is necessary to explore the long‐term effects of melatonin on reproductive health, optimize dosing regimens and investigate its detailed mechanisms, including its role in local hormone modulation and blood flow dynamics within the testes.

One limitation of this study is the relatively short treatment duration, which may not have fully encompassed the complete spermatogenesis cycle in dogs, potentially limiting the ability to observe the full extent of melatonin's effects on reproductive parameters. Additionally, the sample size was limited due to the variability in response among dogs with Alopecia X, which reduced the statistical power and may have affected the ability to detect significant changes in certain parameters. Another important consideration is the observed decrease in the testosterone‐to‐oestradiol ratio in the treatment groups. Although previous studies, such as Salama et al. (2022), have shown that melatonin can influence reproductive hormones and testicular haemodynamics, the clinical relevance of this hormonal shift in our study remains unclear and warrants further investigation (Salama et al. 2022). Moreover, due to international sanctions, measuring melatonin levels in the blood was not feasible, which could have provided more insight into its pharmacokinetics and biological effects.

## 5 Conclusion

This study provides evidence supporting the potential benefits of melatonin in improving reproductive parameters in male dogs, particularly those affected by Alopecia X. The observed dose‐dependent improvements in semen quality and testicular blood flow highlight melatonin's role in enhancing reproductive function. However, the findings also indicate a complex interaction between melatonin dosage and endocrine balance, as reflected in the changes in testosterone‐to‐oestradiol ratios. Although no significant hormonal disruptions were observed, the slight reduction in the ratio in the treatment groups warrants further investigation into its clinical implications. Additionally, the study's relatively short duration and limited sample size may have influenced the observed effects, emphasizing the need for longer term studies that encompass a full spermatogenic cycle. Future research should focus on elucidating the underlying mechanisms of melatonin's action on reproductive hormones, assessing its long‐term safety and evaluating its applicability across different canine breeds and reproductive conditions to establish comprehensive veterinary guidelines.

## Author Contributions

**Arman Abdous**: writing – original draft, resources, data curation. **Mohammad Jokar**: writing – original draft, resources, data curation, formal analysis. **Mehran Farhoodi**: supervision, project administration. **Dariush Shirani**: supervision, methodology. **Fariborz Moayer**: investigation, validation.

## Ethics Statement

The study was conducted in accordance with the ethical standards of the institutional animal care and use committee. Informed consent was obtained from all dog owners prior to the commencement of the study, and all procedures were carried out with the utmost consideration for animal welfare. Approval for the study was granted by the Ethical Review Committee at Islamic Azad University—Karaj Branch (Approval Code: IR.IAU.K.REC.1398.089), dated 2020‐01‐08.

## Conflicts of Interest

The authors declare no conflicts of interest.

### Peer Review

The peer review history for this article is available at https://publons.com/publon/10.1002/vms3.70410.

## Data Availability

The data supporting the findings of this study are available upon reasonable request from the corresponding author.
